# Preoperative Carbohydrate Loading in Pediatric Surgery: A Scoping Review of Current Evidence

**DOI:** 10.12688/f1000research.156172.1

**Published:** 2024-09-24

**Authors:** Yunita Widyastuti, Djayanti Sari, Anisa Fadhila Farid, Amar Rayhan

**Affiliations:** 1 Department of Anesthesiology and Intensive Care, Faculty of Medicine, Public Health and Nursing, Dr. Sardjito General Hospital, Gadjah Mada University, Yogyakarta, Special Region of Yogyakarta, Indonesia

**Keywords:** Preoperative Carbohydrate Loading, Pediatric Surgery, Preoperative Fasting, Perioperative Care

## Abstract

**Introduction:**

Preoperative carbohydrate loading (PCL), part of Enhanced Recovery After Surgery (ERAS) protocols, involves giving carbohydrate-rich liquids before surgery instead of traditional fasting. It improves glucose control, reduces insulin resistance, and enhances patient comfort.

**Methods:**

This scoping review aims to assess the current evidence on the effects and safety of PCL in pediatric surgery. A multi-database search strategy would be used, with eligibility criteria including recent original English articles on pediatric PCL. Data extraction would focus on PCL type, sample sizes, and perioperative outcomes.

**Results:**

The scoping review examined 10 studies on PCL in pediatric surgery, covering various procedures with sample sizes ranging from 18 to 1200 participants. Most studies showed that PCL improved metabolic outcome and reduced postoperative recovery time. However, outcomes like hospital stay length and postoperative complications, such as nausea and vomiting, varied.

**Conclusions:**

PCL in pediatric surgery may stabilize blood glucose, reduce metabolic risks, and enhance recovery, including anxiety reduction.

## 1. Introduction

Preoperative carbohydrate loading (PCL) has emerged as a key component in optimizing surgical outcomes, particularly within the framework of Enhanced Recovery After Surgery (ERAS) protocols. The practice involves administering carbohydrate-rich clear liquids to patients in the hours leading up to surgery, which contrasts with the traditional approach of fasting from midnight. This approach has shown benefits, including improving perioperative glucose control, reducing insulin resistance, and enhancing patient comfort. Additionally, preoperative carbohydrate therapy offers physiological advantages, such as reducing the stress response associated with surgery and contributing to better recovery. While the evidence supporting PCL continues to grow, these studies underline the need for further research to establish standardized protocols and maximize patient outcomes across diverse surgical populations. ^1^^,^^2^

PCL has been increasingly supported by recent studies, particularly in the adult population, for its ability to enhance patient comfort and improve clinical outcomes in elective surgeries. Notably, the use of PCL has shown promise in elderly patients, who often face additional risks during surgery due to their age and comorbidities. In these populations, administering carbohydrate-rich liquids 2–4 hours before surgery has been associated with reduced preoperative thirst and hunger, without adversely affecting insulin resistance (IR) or gastric volume. Moreover, PCL has been linked to significant reductions in postoperative IR, inflammation, and the length of hospital stay in patients undergoing major procedures such as colorectal surgery. ^3^^–^^5^ In pediatric patients, while research is more limited, similar benefits have been observed, including less nausea and reduced gastric contents with no increased aspiration risk during procedures. Further studies are needed to determine the optimal use of carbohydrate loading in various pediatric age groups and conditions. ^6^

## 2. Methods

### 2.1 Research quesition

The scoping review main question is “What is the current evidence on the effects and safety of PCL in pediatric surgery?”.

### 2.2 Search strategy

To conduct a comprehensive scoping review on PCL in pediatric surgery, we used a multi-database approach. The databases to be searched include PubMed (MEDLINE), CINAHL, EMBASE, Cochrane Library, Scopus, and Web of Science. The searchemployed a range of terms such as (“Preoperative Carbohydrate Loading” OR “Preoperative Carbohydrate Supplementation” OR “Preoperative Carbohydrate Administration”) AND (“Pediatric Surgery” OR “Child Surgery” OR “Pediatric Surgical Procedures” OR “Children Surgery”).

### 2.3 Eligiblity criteria

Recent primary original english articles examining PCL in pediatric patients were included from scholarly journals. There were no limitation in study design, surgical procedure and study outcome. Exclusion criteria filtered out articles not related to pediatric populations or those focused exclusively on adult populations or unrelated to PCL. The PRISMA flow chart provides a detailed summary of the study selection process, documenting the number of articles that advanced through each stage of screening.

### 2.4 Data selection and extraction

The selection process will involved screening titles and abstracts to select relevant studies based on inclusion criteria. Full-text articles would be reviewed to confirm relevance and quality. Multiple reviewers ensured accuracy and consistency, resolving discrepancies through discussion or a third reviewer if needed. For each article, data were extracted concerning type of pediatric surgery, sample sizes, type of PCL and control group used, and outcomes measured and results. The outcome results were reported from each article individually, presenting the available information in accordance with the data provided, such as mean, standard deviation (SD), median, interquartile range (IQR), sample size (n), percentage (%), and p-values.

### 2.5 Risk of bias

The authors independently reviewed the methodological quality of the studies using the ‘Risk of bias’ tool, which has undergone modifications and improvements, with an updated version Risk of Bias (RoB) Assessment 2.0 Tools. They evaluated key aspects such as the randomization process, deviations from the intended intervention, missing outcome data, outcome measurement, and selection of reported outcomes. Each study was classified as having low, some concerns, or high risk of bias for each domain. Studies were rated as “low risk” if the information was clear and complete, “high risk” if certain details were missing or suggested a clear risk of bias, and “some concerns” if the data were incomplete.

## 3. Results

**Figure 1.**
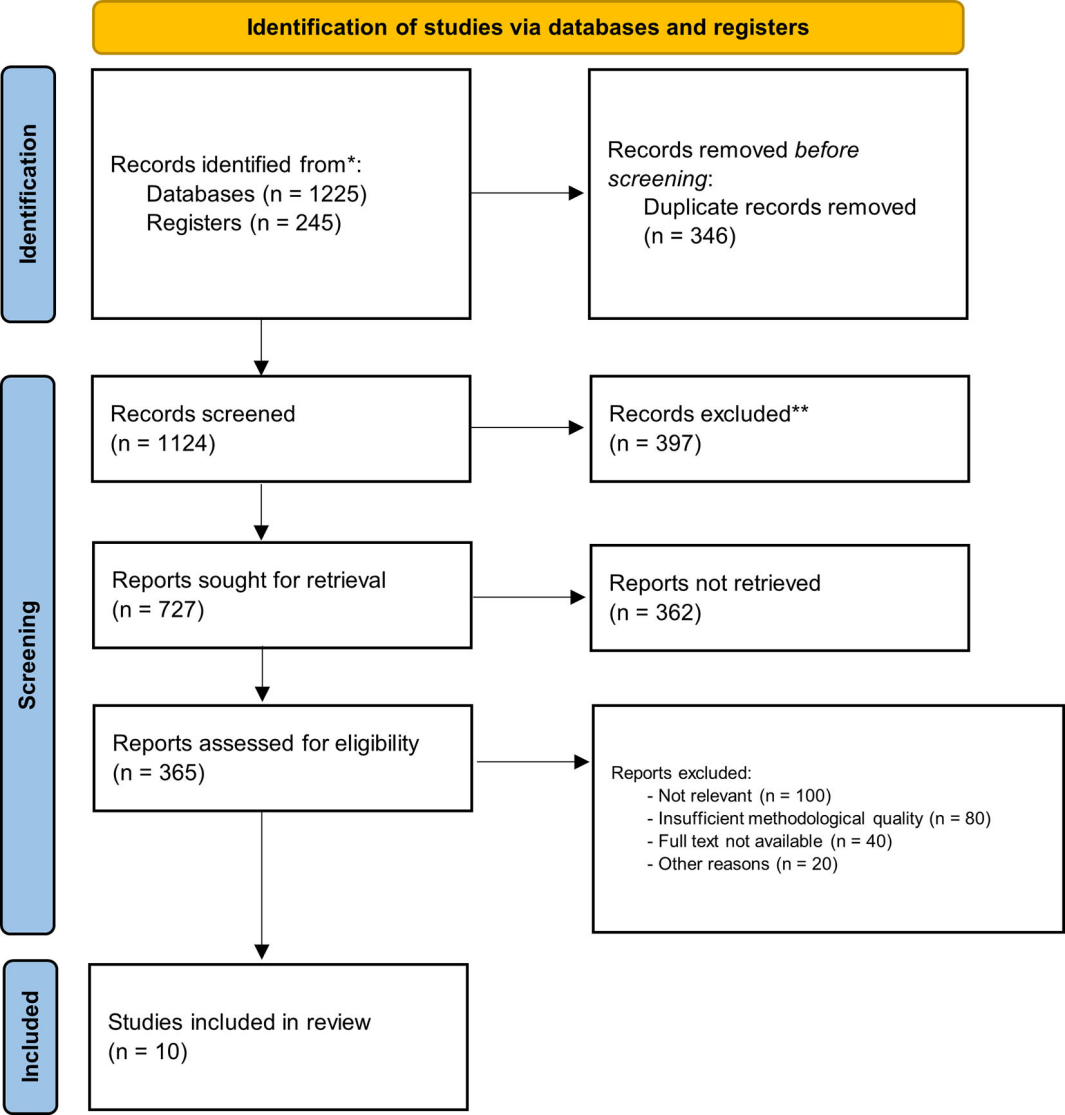
PRISMA flow diagram.

**Figure 2.**
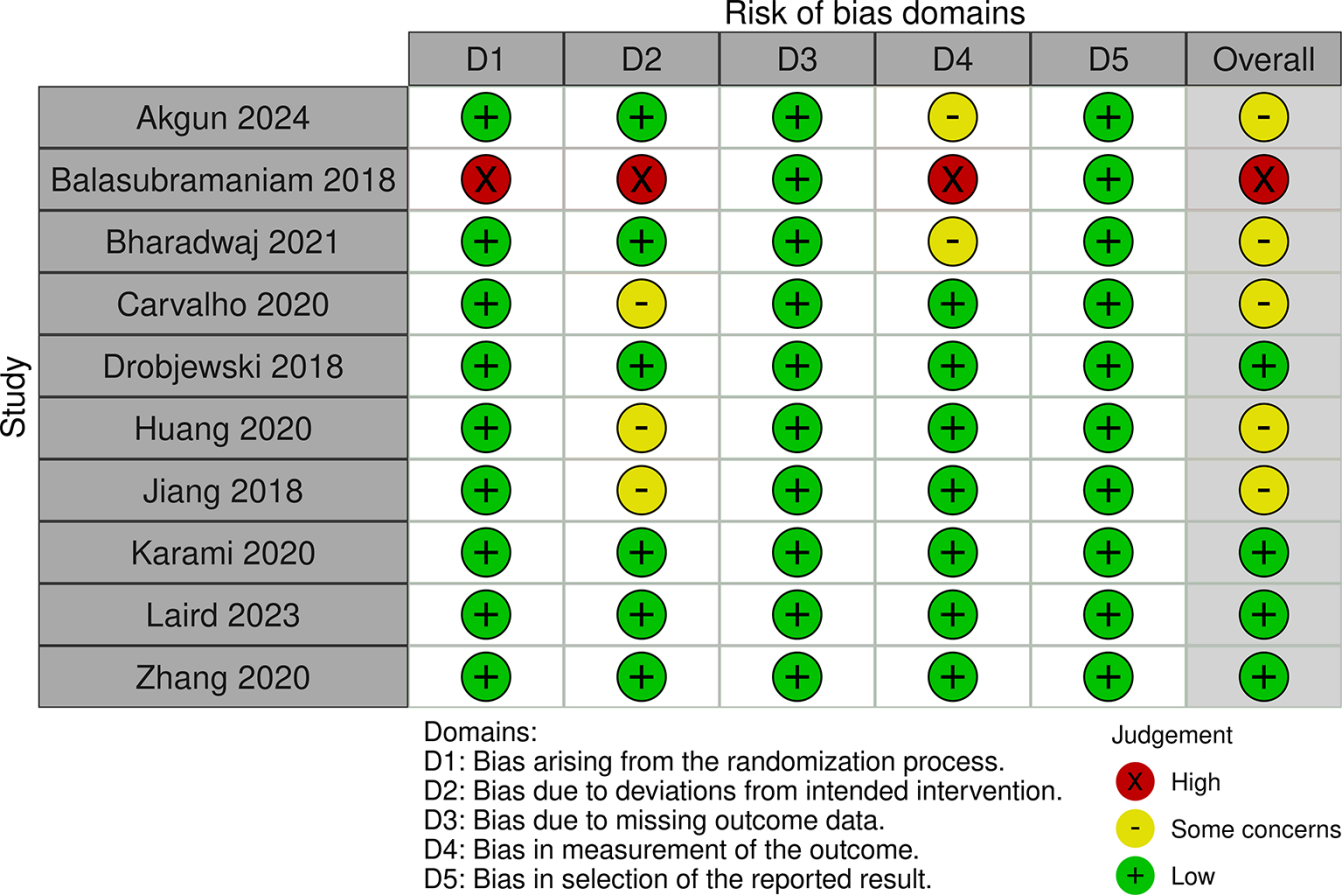
RoB 2.0 traffic light plot.

**Figure 3.**
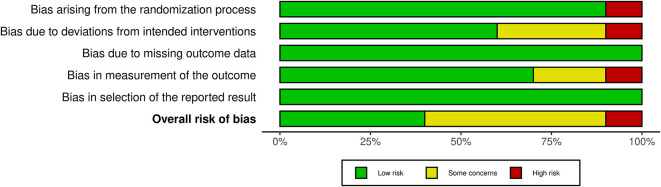
RoB 2.0 summary plot.

The scoping review identified a total of 10 studies that investigated the effects of PCL in pediatric surgery ( Figure 1 and Table 1). The studies included a diverse range of surgical procedures, with most focusing on elective operations such as abdominal, orthopedic, and cardiac surgeries. Sample sizes varied across the studies, ranging from 18 to 1200 participants. Interventions typically involved administering a carbohydrate-rich drink 2-3 hours before surgery, with comparison groups following traditional fasting protocols. The outcomes reported included stomach content, metabolic indicators, incidence of preoperative anxiety and agitation, postoperative complications including nausea and vomiting and length of hospital stay. Additionally, we assessed the risk of bias and found that 4 studies had a low risk, 5 studies had some concerns, and 1 study had a high risk ( Figures 2 and 3).

**Table 1.** Study Characteristics.

No	Author (year)	Study design	Number of patients	Carbohydrate intervention group (n)	Comparison group (n)	Outcome	Outcome results
1	Akgun (2024) ^13^	RCT	90	11 g/100 mL apple juice without fat or protein with 5 mL/kg dose 1 hour before surgery (Group C, n = 30)	5 mL/kg Water 1 hour before surgery (Group W, n =30) and 6 hour fasting group (Group F, n = 30)	1. modified Yale Preoperative Anxiety Scale (m-YPAS) 2. gastric residual fluid volume (GRV) 3. gastric antral cross-sectional area (CSA) 4. 20 ^th^ minute of surgery blood glucose.	1. m-YPAS 1 hour after surgery (Mean±SD; Median [Q1 – Q3]) - Group F = 63.3±16.0; 65 [50–72.0] - Group W = 47.0±17.1; 48.3 [32.9–59.6] - Group C = 31.6±8.5; 28.3 [23.3–37.0] - p value = <0.001 2. GRV, mL (Mean±SD; Median [Q1 – Q3]) - Group F = 18.5±8.2; 16.7 [13.1–20.6] - Group W = 12.9±5.0; 11.8 [9.5–16.5] - Group C = 12.7±6.2; 11.4 [7.7–18.0] - p value = <0.001 3. Antral CSA, ${\mathrm{cm}}^{2}$ (Mean±SD; Median [Q1 – Q3]) - Group F = 4.0±1.9; 3.8 [2.9–4.7] - Group W = 2.7±1.0; 2.5 [2.0–3.4] - Group C = 2.3±1.2; 2.0 [1.5–2.5] - p value = <0.001 4. blood glucose (Mean±SD; Median [Q1 – Q3]) - Group F = 112.8±23.3; 109.0 [102.3–118.5] - Group W = 101.7±14.6; 97.5 [92.0–113.0] - Group C = 90.9±12.8; 90.0 [82.0–102.3] - p value = <0.001
2	Karami (2020) ^12^	RCT	120	5 ml/kg of 20% dextrose solution 2 hours before surgery (Intervention Group, n = 60)	5 ml distilled water (Control Group, n = 60)	1. Perioperative agitation	1. Perioperative agitation (n [%]) - Intervention Group = 6 [10] - Control Group = 33 [55] - p value = 0.001
3	Laird (2023) ^7^	RCT	119	sugar-free cordial drink containing a carbohydrate load (Polyjoule® 24%, 90kcal/100 ml) 2 hours before surgery (Group A, n = 60)	sugar-free cordial and water drink (Group B, n = 59)	Incidence of postoperative nausea, vomiting, pain, and irritability, LOS and preoperative before induction metabolic state (blood glucose, ketone and gases)	1. Postoperative nausea (n [%]) - Group A = 17 [28.3] - Group B = 15 [25.4] - p value = 0.9 2. Postoperative vomiting (n [%]) - Group A = 7 [11.7] - Group B = 9 [15.3] - p value = 0.8 3. Postoperative pain (n [%]) - Group A = 25 [41.7] - Group B = 27 [45.7] - p value = 0.7 4. Postoperative Irritability (n [%]) - Group A = 20 [33.3] - Group B = 14 [23.7] - p value = 0.3 5. Postoperative LOS, minute (Median [Q1 – Q3]) - Group A = 135 [85-235] - Group B = 161 [90-354] - p value = 0.02 6. blood glucose levels, mmol/L (Median [Q1 – Q3]) - Group A = 5.4 [3.3-9.4] - Group B = 4.9 [3.6-6.5] - p value = 0.01 7. blood ketone levels (Median [Q1 – Q3]) - Group A = 0.2 [0-1.4] - Group B = 0.3 [0-1.6] - p value = 0.0003 8. venous blood pH (Median [Q1 – Q3]) - Group A = 7.36 [7.24-7.44] - Group B = 7.37 [7.25-7.44] - p value ≥ 0.9 9. venous blood lactate, mmol/L (Mean [95 % CI]) - Group A = 1.3 [1.25, 1.51] - Group B = 1.0 [0.96, 1.15] - p value ≤ 0.0001
4	Drobjewski (2018) ^15^	RCT	120	5 ml/kg of a lemon-flavoured 0.126 g carbohydrat or 0.5 kcal or 2.15 kJ beverage (PreOp™) 2 hours before endoscopy (Carbohydrate group, n = 60)	Standart fasting, 6 h for solid foods, 4 h for breast milk adn 2 h for clear fluids (Fasting group, n = 60).	Volume and pH of each patient’s stomach content, preoperative thirst and hunger, postoperative nausea and vomiting, and perioperative discomfort ratings	1. Mean volume of gastric content (Mean [SD]), ml/kg 0.01 - Carbohydrate Group = 0.41 [0.28] - Fasting Group = 0.28 [0.27] - p value = 0.01 2. pH of gastric content (Mean [SD]) - Carbohydrate Group = 1.9 [0.5] - Fasting Group = 2.0 [0.6] - p value = not significant 3. Preoperative thirst (n [%]) - Group A = 20 [32] - Group B = 14 [30] - p value = not significant 4. Preoperative hunger (n [%]) - Group A = 18 [30] - Group B = 20 [33] - p value = not significant 5. Postoperative nausea (n [%]) - Group A = 24 [25] - Group B = 6 [10] - p value = 0.028 6. Postoperative vomiting (n [%]) - Group A = 3 [5] - Group B = 1 [2] - p value = not significant
5	Jiang (2018) ^8^	prospective, multi-center, randomized study	1200	10% carbohydrate solution 2 h before anesthesia. (Group B 5 mL/kg, n = 300; Group C 10 mL/kg, n = 300; Group D 15 mL/kg, n = 300)	preoperative fasting at 6 hours before anaesthesia (Group A, n = 300)	Blood glucose, gastric residual, crying ratio perioperatively, hospital stay	1. The blood glucose = significant higher in groups B, C, and D than group A at the time of anesthesia. 2. The gastric residual = no residue in groups A, B, and C. 15 infants in group D had a gastric residual volume. 3. The crying ratio was significantly higher in group A. 4. The length of hospital = not significant different between the groups.
6	Zhang (2020) ^16^	Randomized crossover study	18	5 ml/kg 5% glucose solution or carbohydrate-rich drink (CHO Group, n = 18)	Fasting (GS Group, n = 18)	gastric emptying time (antral CSA and gastric fluid volume), thirst and hunger	1. gastric emptying time, ${\mathrm{cm}}^{2}$ (Mean [95% CI, P Value]) - CHO Group = significantly increased compared to baseline at 10 minutes 2.4 [1.5-3.4, 0.02], 30 minutes 1.1 [0.2-2.1, 0.02], and 60 minutes 1.5 [0.6-2.4, <0.001]. - GS Group = significantly increased compared to baseline at 10 minutes 1.3 [0.6-2.0, 0.02]. 2. gastric fluid volume, ml (Mean Difference [95% CI]) - CHO Group = significantly increased compared to baseline at 10 minutes −0.71 [−1.08 to −0.34], 30 minutes −0.38 [−0.71 to −0.05], and 60 minutes −0.43 [−0.86 to 0.01]. - GS Group = significantly increased compared to baseline at 10 minutes −0.38 [−0.64 to −0.13]. 3. thirst (median [IQR; range]) - CHO Group = significantly increased compared to baseline at 10 minutes 1.5 [0-4.0; 0-10], 30 minutes 2.0 [2.0-4.8; 0-8], and 60 minutes 3.0 [2.0-6.0; 0-8]. - GS Group = significantly increased compared to baseline at 10 minutes 0 [0-0; 0-3], 30 minutes 1.0 [0-2.8; 0-5], and 60 minutes 1.5 [0-3.5; 0-6]. - p value 10 minutes 0.01, 30 minutes 0.02, and 60 minutes 0.01 4. hunger (median [IQR; range]) - CHO Group = significantly increased compared to baseline at 10 minutes 2 [0.3-3.0; 0-5], - GS Group = significantly increased compared to baseline at 10 minutes 4 [3.3-5.0; 0-6] - p value = not significant
7	Huang (2020) ^14^	RCT	351	oral glucose water (10 g of glucose in 100 ml of warm water, 5 ml/kg). 2 hour group (174) vs 1 hour group (170)		Volume of gastric content, preoperative blood glucose, Distribution of gastric volume and pH Pre- and intraoperative adverse reactions	1. Volume of gastric content, ml/kg body weight (Mean ± SD [95% CI, p Value] - 1-h fast group = 0.34±0.35 [0.29–0.39] - 2-h fast group = 0.43±0.33 [0.38–0.48] - p value = 0.011 2. Blood glucose, mmol/L (Mean ± SD [95% CI]) - 1-h fast group = 5.59±1.11 [5.43, 5.76] - 2-h fast group = 6.21±0.78 [6.09, 6.33] - p value = < 0.001 3. Volume of gastric content >0.4 ml/kg of body weight (n [%]) - 1-h fast group = 18 [10.6] - 2-h fast group = 35 [20.1] - p value = 0.014 4. pH of gastric content <2.5 (n [%]) - 1-h fast group = 88 [51.8] - 2-h fast group = 92 [52.9] - p value = not significant 5. Pre- and intraoperative adverse reactions (n [%]) - 1-h fast group = Crying, 68 [40]; Thirst, 35 [20.6]; Hypoxia, 9 [5.3]; Vomiting, 7 [4.1]; Pulmonary Aspiration, 3 [1.8]; Heart Failure, 2 [1.2]. - 2-h fast group = Crying, 90 [51.7]; Thirst, 58 [33.3]; Hypoxia, 20 [11.5]; Vomiting, 6 [3.4]; Pulmonary Aspiration, 3 [1.7]; Heart Failure, 3 [1.7]. - p value = Crying, 0.029; Thirst, 0.008; Hypoxia, 0.039; Vomiting, 0.745; Pulmonary Aspiration, 0.647; Heart Failure, 0.511.
8	Carvalho (2020) ^9^	RCT	40	12.5% maltodextrin diluted in 150 mL of water (CHO Group, n = 19)	Fasting (Fasting Group, n = 21)	albumin, interleukin 6 (IL-6), blood glucose, insulin and C-reactive protein (CRP). Insulin resistance with HOMA IR Index	1. albumin (Mean±SD) - Fasting Group = preoperative, 4.08±0.39; postoperative, 3.82 ± 0.48 - CHO Group = preoperative, 4.12 ± 0.29; postoperative, 3.77 ± 0.29 - p value = preoperative, 0.94; postoperative, 0.53 2. IL-6 - Fasting Group = preoperative, 1.5 ± 2.6; postoperative, 3.82 ± 0.48 - CHO Group = preoperative, 2.0 ± 2.3; postoperative, 1.5 ± 2.0 -p value = preoperative, 0.98; postoperative, 0.41 3. blood glucose - Fasting Group = preoperative, 88 ± 16; postoperative, 91 ± 34 -CHO Group = preoperative, 86 ± 9; postoperative, 93 ± 24 - p value = preoperative, 0.32; postoperative, 0.60 4. insulin - Fasting Group = preoperative, 3.09 ± 6.34; postoperative, 91 ± 34 - CHO Group = preoperative, 4.90 ± 4.52; postoperative, 4.55 ± 3.43 - p value = preoperative, 0.69; postoperative, 0.78 5. CRP - Fasting Group = preoperative, 3.60 ± 7.60; postoperative, 3.53 ± 7.75 - CHO Group = preoperative, 0.53 ± 0.59; postoperative, 0.49 ± 0.53 - p value = preoperative, 0.05; postoperative, 0.02 6. HOMA IR - Fasting Group = preoperative, 0.86 ± 2.05; postoperative, 91 ± 34 - CHO Group = preoperative, 1.57 ± 1.86; postoperative, 1.13 ± 1.05 - p value = preoperative, 0.49; postoperative, 0.37 7. PCR/albumin - Fasting Group = preoperative, 0.89 ± 1.86; postoperative, 0.91 ± 1.97 - CHO Group = preoperative, 0.13 ± 0.15; postoperative, 0.13 - ± 0.15 - p value = preoperative, 0.03; postoperative, 0.08
9	Bharadwaj (2021) ^10^	RCT	101	5 mL/kg Body Weight (BW) of pulp-free clear apple juice (Tropicana (100 mL pack)-containing 12% sugar, Tropicana Products, Inc., division of PepsiCo, Inc., Chicago, USA), 2 hours prior to induction of anaesthesia (Study Group, n = 50)	Standard ASA fasting guidelines (6-4-2 regimen) (Control Group, n = 51)	Preoperative and postoperative of UMSS and behavior score. child parent separation score and mask acceptance score. Time to attain MAS, to ask for oral intake and time for attaining the discharge criteria. Random blood sugar with 2 minutes interval in 51 minute	1. Time to attain MAS of >9, minutes (Mean±SD [Range]) - Study Group = 18.70±10.19 [5-40] - Control Group = 16.86±6.85 [0-30] - p value = 0.007 2. Time to ask for oral intake, minutes (Mean±SD [Range]) - Study Group = 49.40±31.8 [10-120] - Control Group = 25.88±16.93 (5-90] - p value = 0.0001 3. Time for attaining the discharge criteria, minutes (Mean±SD [Range]) - Study Group = 35.5±14.11 [15-6] - Control Group = 38.04±13.71 [20-60] - p value = 0.841 4. Preoperative UMSS (Median [Range]) - Study Group = 0 (0-2) - Control Group = 1 (0-3) - p value = 0.001 5. Postoperative UMSS (Median [Range]) - Study Group = 2 [0-3] - Control Group = 2 [0-3] - p value = 0.305 6. Preoperative behaviour score (Median [Range]) - Study Group = 3 [1-4] - Control Group = 3 [1-4] - p value = 0.667 7. Postoperative behaviour score (Median [Range]) - Study Group = 3.5 [1-4] - Control Group = 3 [1-4] - p value = 0.412 8. Child-parent separation score (Median [Range]) - Study Group = 2 (1-3) - Control Group = 2 (1-3) - p value = 0.96 9. Mask acceptance score (Median [Range]) - Study Group = 3 (1-4) - Control Group = 3 (1-4) - p value = 0.659 10. Random blood sugar, mg/dL (Median [IQR]) - Study Group = 70 [60-79] - Control Group = 90 [85-98] - p value = 0.005
10	Balasubramaniam (2018) ^11^	RCT	120	2 ml per kg of body weight of 10% Dextrose water as oral feeds half hour before the expected time of start of anaesthesia (Group B, n = 60)	fasting according to ASA guidelines preoperatively (Group A, n = 60)	Preoperative blood sugar, hypoglycemia	1. Preoperative blood glucose concentrations (Mean±SD [Range]) - Group A = 64.08 ± 25.37 - Group Group B = 102.5 ± 16.97 - p value ≤ 0.0001 2. Hypoglycemia (n [%]) - Group A = 39 (65) - Group Group B = 14 (23)

### 3.1 Metabolic effects

The research on PCL demonstrates its significant impact on stabilizing blood glucose levels and enhancing metabolic control before pediatric surgery. In studies comparing carbohydrate-loaded patients with those in control or fasting groups, those who received carbohydrates generally had higher, yet stable, blood glucose levels. For example, patients in the carbohydrate group showed a median blood glucose level of 5.4 mmol/L, which was slightly higher than that of the placebo group. This suggests that PCL helps maintain energy reserves during the perioperative period, reducing the risk of hypoglycemia and providing metabolic benefits. ^7^ Additionally, carbohydrate-loaded patients exhibited fewer abnormal ketone levels, indicating better overall metabolic stability, although a slight increase in lactate levels was noted. These findings underscore the potential of PCL to enhance patient outcomes by ensuring more consistent blood glucose levels before surgery. ^8^

Further research supports these benefits by showing that PCL not only stabilizes blood glucose levels but also reduces the likelihood of hyperglycemia and hypoglycemia. In one study, fasting patients were more prone to hyperglycemia, with 21% exceeding 99 mg/dL, while none of the patients in the carbohydrate group experienced hyperglycemia. Both groups had similar insulin levels and Homeostatic Model Assessment for IR (HOMA-IR) Index data before and after the operation, indicating that carbohydrate loading helps prevent hyperglycemia without affecting insulin resistance. ^9^ Another study found significantly lower random blood sugar levels in the carbohydrate group compared to the control group, where higher glucose levels were trending. This suggests that carbohydrate loading effectively prevents the sharp fluctuations in blood glucose that can pose risks during surgery. ^10^ Moreover, the fasting group was found to experience more frequent signs of hypoglycemia, such as excessive crying, sweating, and irritability, highlighting the discomfort and potential risks associated with fasting. ^11^ Overall, the evidence points to PCL as a valuable strategy for improving preoperative care by maintaining blood glucose stability, reducing metabolic risks, and enhancing patient comfort.

### 3.2 Preoperative anxiety and agitation

The studies collectively highlight the positive impact of PCL and varying fasting durations on reducing perioperative anxiety, agitation, and crying in pediatric patients. One study found that children who received PCL were significantly calmer, with 90% being quiet and relaxed compared to only 11.7% in a control group, indicating a notable decrease in postoperative agitation. ^12^ Another investigation showed that preoperative anxiety levels were markedly lower in children who consumed carbohydrate fluids, as measured by the modified Yale Preoperative Anxiety Scale (m-YPAS), compared to those who fasted. ^13^ While one randomized clinical trial found no significant differences in behavior scores, separation anxiety, or mask acceptance between PCL and fasting groups, other research demonstrated that PCL significantly reduced preoperative crying, likely due to its ability to alleviate thirst and hunger. This reduction in crying not only conserves energy but also prevents gastrointestinal flatulence, which can interfere with surgical procedures. ^8^^,^^10^ Additionally, a comparison of fasting durations revealed that a 1-hour fasting period resulted in fewer instances of crying than a 2-hour fast, supporting the effectiveness of shorter fasting intervals combined with PCL. ^14^ Overall, the studies emphasize the benefits of PCL in minimizing anxiety, agitation, and crying, thereby improving the preoperative experience for pediatric patients.

### 3.3 Stomach content

The studies provide a comprehensive analysis of how PCL and different fasting durations impact gastric content, gastric emptying, and the associated risks of pulmonary aspiration. Significant differences were observed in antral cross-sectional area (CSA) and gastric residual volume (GRV) between groups, with both PCL and water intake leading to reduced gastric content compared to fasting. ^13^ This reduction is further emphasized by findings that PCL led to a 68% decrease in gastric content compared to fasting, highlighting its effectiveness in minimizing gastric volume. ^15^ Temporal changes in antral CSA indicate that PCL causes a significant increase within the first 10 minutes post-ingestion, which then returns to baseline by 90 minutes, underscoring the importance of timing in PCL administration. ^16^

In terms of safety, administering PCL at doses up to 10 mL/kg does not increase gastric residuals in adults, children, or infants, thereby reducing the risk of regurgitation and aspiration. The absence of significant gastric residuals across these groups suggests that PCL is well-tolerated and does not compromise gastric emptying. ^8^ Additionally, a comparison of fasting durations reveals that a 1-hour fast results in lower gastric content volume than a 2-hour fast, without increasing the risk of low gastric pH or the simultaneous presence of large gastric volumes and low pH, which are key factors in pulmonary aspiration risk. ^14^ Collectively, these studies underscore the efficacy and safety of PCL in reducing gastric content and improving patient outcomes by lowering the risk of aspiration during surgery.

### 3.4 Postoperative complications

PCL indicate that it generally does not lead to significant postoperative complications compared to fasting or placebo groups. For instance, the incidence of nausea, vomiting, and pain were similar between groups, with no significant differences observed in postoperative anti-emetic use or pain severity. In one study, children who received carbohydrates were more likely to experience severe nausea in the first postoperative hour, but this difference did not persist beyond that time. Additionally, there was no difference in the time taken to meet discharge criteria between those who received carbohydrates and those who did not. ^7^^,^^10^^,^^11^

Other studies reported no complications during the study period, and PCL was associated with reduced incidences of crying, thirst, and hypoxia compared to longer fasting durations. Moreover, no adverse reactions such as pulmonary aspirations, heart failure, or severe hunger were observed in the groups receiving carbohydrates. ^14^^,^^16^ These findings suggest that PCL is generally safe and does not increase the risk of postoperative complications, while it may offer some benefits in reducing discomfort after surgery.

### 3.5 Length of hospital stay

The study results indicate that PCL has a potential impact on the length of stay (LOS) in a hospital setting. One study found a significant reduction in the median LOS for children in the carbohydrate group (135 minutes) compared to the placebo group (161 minutes), suggesting that carbohydrate loading may enhance postoperative recovery and reduce hospital stay. However, the clinical relevance of this 26-minute reduction is debatable, and the study suggests that maintaining adequate hydration alone, regardless of carbohydrate intake, could improve postoperative recovery. It was also noted that the effect was more pronounced in morning surgeries, potentially influencing future clinical guidelines for preoperative hydration. ^7^ Another study, however, found no significant difference in the length of hospital stay between groups receiving PCL and those who did not. ^8^ This inconsistency highlights the need for further research to better understand the factors influencing LOS and the role of PCL in pediatric surgical recovery.

## 4. Discussion

The ASA guideline emphasizes the importance of adhering to recommended fasting durations to avoid adverse patient and clinical outcomes. Prolonged fasting, whether due to unclear or extended fasting policies, can increase preoperative thirst, hunger, anxiety, and discomfort, as well as lead to dehydration and hypotension during anesthesia induction. Although recent European and Canadian guidelines suggest reducing clear liquid fasting to 1 hour in children, the ASA task force refrains from making a strong recommendation due to limited and low-quality evidence. ^17^^–^^20^

In this context, PCL emerges as a promising intervention to address some of these challenges, particularly in pediatric populations. The findings from this scoping review suggest that PCL may offer several benefits, including stabilizing blood glucose levels, reducing metabolic risks, and improving perioperative outcomes. Studies consistently demonstrated that PCL helps maintain higher yet stable blood glucose levels, thereby reducing the risk of hypoglycemia during the perioperative period. This metabolic stability is crucial for pediatric patients, ensuring that energy reserves are maintained, potentially leading to better recovery outcomes.

However, the review also highlighted variability in outcomes such as LOS and the incidence of postoperative complications. While some studies reported a significant reduction in LOS for patients receiving PCL, others found no significant differences between the PCL and control groups. This inconsistency suggests that the effectiveness of PCL may be influenced by factors like the timing of surgery, the type of surgical procedure, and individual patient characteristics.

Moreover, although PCL generally did not increase the risk of postoperative complications, the extent of its benefits varied across different studies. Some studies reported reductions in postoperative complications such as nausea, vomiting, and pain, while others did not. These mixed results highlight the need for further research to better understand the specific conditions under which PCL provides the most benefit.

In evaluating the risk of bias among the included studies, we found that four studies had a low risk, five studies had some concerns, and one study had a high risk. This variability in study quality may partially explain the inconsistent findings related to PCL outcomes, such as the LOS and the incidence of postoperative complications. While some studies reported significant benefits from PCL, others did not, indicating that the effectiveness of PCL might be influenced by factors like surgical timing, procedure type, and patient characteristics.

Future research should therefore prioritize the optimization of PCL protocols and evaluate its effects across a wider spectrum of surgical procedures. It is particularly crucial to investigate the most effective practices for PCL in pediatric populations, where the current body of evidence is notably limited. Research efforts should focus on identifying the optimal timing, dosage, and composition of PCL to fully harness their potential benefits. Furthermore, certain outcomes, including advanced metabolic indicators such as insulin levels, HOMA-IR, blood analysis, and inflammation biomarkers like IL-6, have been inadequately explored in existing studies. Addressing these gaps in knowledge will be essential for refining preoperative care practices, with the ultimate goal of improving patient outcomes and reducing the adverse effects associated with prolonged fasting.

## 5. Conclusions

PCL in pediatric surgery may stabilize blood glucose, reduce metabolic risks, improve recovery, and minimize postoperative complications. However, evidence on safety and outcomes, such as hospital stay length and postoperative complications, remains inconsistent, highlighting the need for further research to refine PCL protocols for pediatric care.

### Ethics and consent

Ethical approval and consent were not required.

## Data Availability

No underlying data are associated with this article. No extended data are associated with this article. Open Science Framework: Preoperative Carbohydrate Loading in Pediatric Surgery: A Scoping Review of Current Evidence, DOI: https://doi.org/10.17605/OSF.IO/KPNV5. ^21^ Data are available under the terms of the Creative Commons Zero “No rights reserved” data waiver (CC0 1.0 Public domain dedication).
