# Supplementary material for: Preoperative Carbohydrate Loading in Pediatric Surgery: A Scoping Review of Current Clinical Trials
Source: F1000Res. 2024 Dec 23;13:1089. Originally published 2024 Sep 24. [Version 3] doi: 10.12688/f1000research.156172.3 (PMC11822248; doi:10.12688/f1000research.156172.3)
Supplement: Peer review history

# Reviewer report: Reviewer response for version 3

**DOI:** 10.5256/f1000research.176192.r361303

**Published:** 2025-02-12

**Authors:** Kalyani Patil

**Affiliations:** 1 Anaesthesia and Pain Medicine., Bharati Hospital, Pune, Maharashtra, India; 2 Bharati Vidyapeeth (Deemed to be) University Medical College, Pune, India

**Major:**

 The search strategy is well described and includes all important databases.

 However there are no details of how the results of all studies were statistically analysed for the common outcomes.

 The results only mention the number of studies having a particular finding.

 There is no detailed mention of a comprehensive quantitative analysis of a particular outcome.

 Forest plot can be included for common and important outcomes as it would make the article more relevant and the findings more objective. 

 The limitation mentions "publication bias" may exist. I suggest that the authors analyse the publication bias using a funnel plot. It would definitely add more value to the article. 

**Minor:**

 The article is relevant and has attempted well to answer the research gap on a topic that is so pertinent in paediatric anaesthesia and would bring a positive change in management and patient outcome.

 The objective is clearly stated.

Are the rationale for, and objectives of, the Systematic Review clearly stated?

Yes

Is the statistical analysis and its interpretation appropriate?

No

If this is a Living Systematic Review, is the ‘living’ method appropriate and is the search schedule clearly defined and justified? (‘Living Systematic Review’ or a variation of this term should be included in the title.)

Not applicable

Are sufficient details of the methods and analysis provided to allow replication by others?

Partly

Are the conclusions drawn adequately supported by the results presented in the review?

Yes

Reviewer Expertise:

Ultrasound guided Regional Anaesthesia; Perioperative Care, Simulation, Obstetric anaesthesia.

I confirm that I have read this submission and believe that I have an appropriate level of expertise to confirm that it is of an acceptable scientific standard, however I have significant reservations, as outlined above.

# Reviewer report: Reviewer response for version 3

**DOI:** 10.5256/f1000research.176192.r357029

**Published:** 2025-01-13

**Authors:** Maliwan Oofuvong

**Affiliations:** 1 Prince of Songkla University, Songkhla, Thailand

It is useful systemic review for application of fasting time with carbohydrate drink in pediatric surgery.

 The result can be applied for clinical practice if the clearer methodology could be addressed.

 1. What is the definition of preoperative carbohydrate loading? It sounds to have various of PCL intervention in the review: 

 Oral vs intravenous and carbohydrate vs dextrose in the intervention group (In Table 1 for 11 paper reviews). How many papers that intravenous intervention were given? Should the intervention confine only carbohydrate oral drink according to the title?

 2. What was the age range included? The age range should be included in the Method section.

 3. Result in page 13, #3 mentioned that "The studies included a diverse range of surgical procedures, with most focusing on elective operations such as abdominal, orthopedic, and cardiac surgeries". Why the authors included cardiac surgery in the systemic review since cardiac surgery encountered with hemodynamic instability, potential higher blood sugar and increase metabolic risk from cardiopulmonary bypass. It may induce the bias of result among intervention and control if different types of cardiac surgery were performed among groups and result from cardiac surgery may be incomparable with non cardiac surgery. How many studies of cardiac surgery had been included in the review?

 4. # 3.5 Length of hospital stay (LOS)

 Only the study of Laird (ref 8) reported the difference of LOS 26 min (135 vs 161 min) in day case surgery. Should the review separate between day case surgery or inpatient surgery? How many papers of day case surgery and inpatient surgery? 

 Minor comments

 1. Figure 1 PRISMA flow diagram, at the screening process, reports assessed for eligibility of 396, reports excluded in total 249 papers, so reports should be remained for review 147, how come only 11 studies were included in the review.

 2. According to paper 7, Huang (2020), study design of RCT, there was no information in the column of comparison group.

Are the rationale for, and objectives of, the Systematic Review clearly stated?

Yes

Is the statistical analysis and its interpretation appropriate?

Yes

If this is a Living Systematic Review, is the ‘living’ method appropriate and is the search schedule clearly defined and justified? (‘Living Systematic Review’ or a variation of this term should be included in the title.)

Not applicable

Are sufficient details of the methods and analysis provided to allow replication by others?

Partly

Are the conclusions drawn adequately supported by the results presented in the review?

Partly

Reviewer Expertise:

Pediatric and cardiac anesthesia.

I confirm that I have read this submission and believe that I have an appropriate level of expertise to confirm that it is of an acceptable scientific standard, however I have significant reservations, as outlined above.

# Reviewer report: Reviewer response for version 3

**DOI:** 10.5256/f1000research.176192.r351979

**Published:** 2024-12-31

**Authors:** Vibhavari Naik, Abhijit Nair

**Affiliations:** 1 Department of Oncoanaesthesia, Pain and Palliative Medicine, Basavatarakam Indo-American Cancer Hospital and Research Institute, Hyderabad, India; 2 Ibra Hospital, Ibra, Ash Sharqiyah North Governorate, Oman

We are happy that authors have considered our suggestions and corrections. We commend the authors for undertaking a review on such a relevant and impactful topic. This review's primary strength lies in its comprehensive exploration of the effects of preoperative carbohydrate loading on metabolic parameters, gastric volume, and psychological comfort. The authors have clearly articulated the study's limitations and proposed valuable directions for future research. We recommend the acceptance of this article for indexing.

Are the rationale for, and objectives of, the Systematic Review clearly stated?

Yes

Is the statistical analysis and its interpretation appropriate?

Partly

If this is a Living Systematic Review, is the ‘living’ method appropriate and is the search schedule clearly defined and justified? (‘Living Systematic Review’ or a variation of this term should be included in the title.)

Not applicable

Are sufficient details of the methods and analysis provided to allow replication by others?

Partly

Are the conclusions drawn adequately supported by the results presented in the review?

No

Reviewer Expertise:

Vibhavari Naik - oncoanaesthesia, paediatric anaesthesia, vascular access; Abhijit Nair - statistics and research methodology, systematic reviews

We confirm that we have read this submission and believe that we have an appropriate level of expertise to confirm that it is of an acceptable scientific standard.

# Reviewer report: Reviewer response for version 2

**DOI:** 10.5256/f1000research.173793.r343632

**Published:** 2024-12-12

**Authors:** Vibhavari Naik, Abhijit Nair

**Affiliations:** 1 Department of Oncoanaesthesia, Pain and Palliative Medicine, Basavatarakam Indo-American Cancer Hospital and Research Institute, Hyderabad, India; 2 Ibra Hospital, Ibra, Ash Sharqiyah North Governorate, Oman

We thank authors for considering the suggestions and making changes to the draft. We have a few unaddressed concerns and would request the authors to consider them to improve the clarity and quality of the article.

 1. The introduction part of abstract can be specific to mention the knowledge gap of 'preoperative carbohydrate loading in children' that the authors intend to address or why they have considered to conduct this topic for review.

 2. The last line of Introduction section of the main article ‘Further studies are needed….’ is inappropriate for Introduction section. Introduction should end with authors mentioning why they conducted this scoping review or what this scoping review aims to address.

 3. In Search strategy, ‘Databases to be searched include’ can be replaced by ‘Databases searched included’

 4. In results section, ‘An additional’ needs to be removed. And {number of excluded records]’ should be replaced by the actual number. ‘The scoping review……in paediatric surgery’ is duplicated. 

 5. Table legends should include the short forms used in the tables.

Are the rationale for, and objectives of, the Systematic Review clearly stated?

Yes

Is the statistical analysis and its interpretation appropriate?

Partly

If this is a Living Systematic Review, is the ‘living’ method appropriate and is the search schedule clearly defined and justified? (‘Living Systematic Review’ or a variation of this term should be included in the title.)

Not applicable

Are sufficient details of the methods and analysis provided to allow replication by others?

Partly

Are the conclusions drawn adequately supported by the results presented in the review?

No

Reviewer Expertise:

Vibhavari Naik - oncoanaesthesia, paediatric anaesthesia, vascular access; Abhijit Nair - statistics and research methodology, systematic reviews

We confirm that we have read this submission and believe that we have an appropriate level of expertise to confirm that it is of an acceptable scientific standard, however we have significant reservations, as outlined above.

# Reviewer report: Reviewer response for version 1

**DOI:** 10.5256/f1000research.171447.r327893

**Published:** 2024-10-18

**Authors:** Vibhavari Naik, Abhijit Nair

**Affiliations:** 1 Department of Oncoanaesthesia, Pain and Palliative Medicine, Basavatarakam Indo-American Cancer Hospital and Research Institute, Hyderabad, India; 2 Ibra Hospital, Ibra, Ash Sharqiyah North Governorate, Oman

**Overall** – The authors have selected a pertinent topic for a scoping review. Although the concept and components of ERAS have been adopted from adult practice, evidence needs to be established to justify its role in paediatric care. In this article, the authors have evaluated the evidence regarding the use of preoperative carbohydrate loading as a component of ERAS in children.

 The authors mention this as a scoping review but the methodology has overlapped with a systematic review. The inclusion of a risk of bias assessment in this study suggests a more rigorous approach typically used with systematic reviews. This being a scoping review authors should intend to provide an overview of the existing evidence including gaps in literature and determine if systematic review is required. Literature review for a scoping review apart from RCTs, should also include case reports, case series, observational studies, guidelines, blogs, etc. Alternatively, since most of the included studies are RCTs, authors could choose to perform a qualitative systematic review and a possible quantitative meta-analysis. But this would require following the stringent PRISMA methodology including study registration.

**Title** - is clear and informative and includes the mention of scoping review

**Abstract **- is structured and conveys the gist of the review. The introduction part of the abstract could end with the objective of the review. In the methods section of abstract, future tense phrases like ‘would be used’ and ‘would focus’ could be changed to past tense like ‘was used’ and ‘was focused’. The authors mention ‘recent’ articles but have not explained what recent means.

**Introduction **– Authors could write introduction more crisply by avoiding repetition of enhancing patient comfort and improving outcomes; and reducing the content on adult population. It broadly could include what is available in literature about paediatric preoperative carbohydrate loading and end with what this review aims to achieve.

**Methods** – The PRISMA-ScR reporting guidelines could be accessed at Open Science Framework platform. But the authors have not mentioned if the protocol for the review exists, registration of the protocol and the link to the same. 

 A formal risk of bias assessment may not be necessary for scoping reviews. 

 Future tense of methodology needs to be changed to past tense throughout the methods section. Ex. The selection process will involved, Full-text articles would be reviewed, etc.

 Eligibility criteria – includes the word ‘recent’ the time duration of which has not been explained. 

 The authors must provide truncations of the search results from various databases in a supplementary file. They could present the full electronic search strategy for at least 1 database, including any limits used, such that it could be repeated. Authors could include the date of database search.

 Figure 1 PRISMA flow diagram mentions - Records excluded** Authors could mention  what two asterisks stand for in the footnote of the figure mentioning the reason for exclusion. Authors mention ‘Reports not retrieved’ but have not mentioned the reason for non-retrieval. Authors mention 240 reports excluded after assessing for eligibility from 365 reports. However only 10 articles are included in review. Authors need to mention the reasons for excluding 115 articles.

 Clarity on how many reviewers contributed to data charting will be useful instead of mentioning ‘multiple’.

**Results** – Table 1 legends should include expansion of short forms used in the table.

 Length of stay – though authors mention inconsistency in results, the results appear to be consistent in studies not demonstrating clinically relevant difference amongst the groups. This could be restructured.

 Carvalho in their abstract mention - Four patients (21%) in the fasting group but none in the CHO group were hyperglycemic before surgery (p = 0.04). This has been missed while compiling this review.

 We have found a few relevant studies which were not included in this review. Could authors provide an explanation for the same?

- Gawecka A, Mierzewska-Schmidt M. Tolerance of, and metabolic effects of, preoperative oral carbohydrate administration in children - a preliminary report. Anaesthesiol Intensive Ther. 2014 Apr-Jun;46(2):61-4. 
- Bauer JM, Trask M, Coughlin G, Gopalan M, Gupta A, Yaszay B, Yang S, Grigg E. Pre-operative carbohydrate drink in pediatric spine fusion: randomized control trial. Spine Deform. 2024 Sep;12(5):1283-1287. 

**Discussion** – Authors mention that - Studies consistently demonstrated that PCL helps maintain higher yet stable blood glucose levels. However, Bharadwaj et al show lower glucose levels in children receiving PCL. The effect of PCL on glucose levels could be rewritten as inconsistent. Authors could highlight and explore the inconsistency in glucose levels after PCL and identify if any factors like duration of fasting affected it.

 In Laird et al. difference of ketones by 0.1 and lactates by 0.3 mmol/L with overlapping ranges though may be statistically significant, the clinical relevance of such small differences may not be relevant. Authors could highlight such trivial ‘statistically significant’ results across all the articles included and explore the clinical relevance in the discussion of this review.

 Authors could include a mention of recently published meta-analysis on the topic and compare results - Li Y, Chen L, Su Y, Zhang X. Preoperative Oral Carbohydrates for Children: A Meta-Analysis and Systematic Review. Biol Res Nurs. 2024 Oct;26(4):624-635. 

 Scoping reviews can have the following biases: language, search, data extraction, reporting, reviewer, publication, and selection bias. Authors need to mention these in the limitations including steps taken to mitigate them.

 Few areas of spelling mistakes and improvement of grammar would be required.

Are the rationale for, and objectives of, the Systematic Review clearly stated?

Yes

Is the statistical analysis and its interpretation appropriate?

Partly

If this is a Living Systematic Review, is the ‘living’ method appropriate and is the search schedule clearly defined and justified? (‘Living Systematic Review’ or a variation of this term should be included in the title.)

Not applicable

Are sufficient details of the methods and analysis provided to allow replication by others?

Partly

Are the conclusions drawn adequately supported by the results presented in the review?

No

Reviewer Expertise:

Vibhavari Naik - oncoanaesthesia, paediatric anaesthesia, vascular access; Abhijit Nair - statistics and research methodology, systematic reviews

We confirm that we have read this submission and believe that we have an appropriate level of expertise to confirm that it is of an acceptable scientific standard, however we have significant reservations, as outlined above.

## References

[1] : Metabolic and Inflammatory Benefits of Reducing Preoperative Fasting Time in Pediatric Surgery. *Rev Col Bras Cir* .2020;47: 10.1590/0100-6991e-20202353 e20202353 10.1590/0100-6991e-20202353 32578813

[2] : Tolerance of, and metabolic effects of, preoperative oral carbohydrate administration in children - a preliminary report. *Anaesthesiol Intensive Ther* .2014;46(2) : 10.5603/AIT.2014.0013 61-4 10.5603/AIT.2014.0013 24858963

[3] : Preoperative Oral Carbohydrates for Children: A Meta-Analysis and Systematic Review. *Biol Res Nurs* .2024;26(4) : 10.1177/10998004241253536 624-635 10.1177/10998004241253536 38860320

# Response

**Published:** 2024-11-05

**Authors:** Amar Rayhan

Thank you for your insightful feedback and constructive comments on our manuscript. We appreciate the opportunity to improve our work and have made significant revisions based on your suggestions. In response, we have made the following adjustments:

**Overall**

Comment from reviewer :

 The authors have selected a pertinent topic for a scoping review. Although the concept and components of ERAS have been adopted from adult practice, evidence needs to be established to justify its role in paediatric care. In this article, the authors have evaluated the evidence regarding the use of preoperative carbohydrate loading as a component of ERAS in children

Respond:

We already added the narrative review about ERAS in pediatric including Preoperative Carbohydrat loading as part of ERAS in pediatric

Comment from reviewer :

 The authors mention this as a scoping review but the methodology has overlapped with a systematic review. The inclusion of a risk of bias assessment in this study suggests a more rigorous approach typically used with systematic reviews. This being a scoping review authors should intend to provide an overview of the existing evidence including gaps in literature and determine if systematic review is required. Literature review for a scoping review apart from RCTs, should also include case reports, case series, observational studies, guidelines, blogs, etc. Alternatively, since most of the included studies are RCTs, authors could choose to perform a qualitative systematic review and a possible quantitative meta-analysis. But this would require following the stringent PRISMA methodology including study registration

Respond:

 Thank you for your feedback, according the scope and methodology we have revised some parts:

1. The title and eligibility criteria have been revised to **"Preoperative Carbohydrate Loading in Pediatric Surgery: A Scoping Review of Current Clinical Trials"** to clarify the focus and methodology of the study. This adjustment emphasizes our intent to provide an overview of existing evidence while addressing gaps in the literature surrounding the topic.
2. Our scoping review now specifies clinical trials research, prompting us to revise the methodology. The RoB (Risk of Bias) tool from Cochrane was chosen to evaluate the critical appraisal of included clinical studies.

**Title**

Comment from reviewer :

****- is clear and informative and includes the mention of scoping review

Respond:

 Thank you very much

**Abstract**

Comment from reviewer :

 is structured and conveys the gist of the review. The introduction part of the abstract could end with the objective of the review. In the methods section of abstract, future tense phrases like ‘would be used’ and ‘would focus’ could be changed to past tense like ‘was used’ and ‘was focused’. The authors mention ‘recent’ articles but have not explained what recent means

Respond:

 Thank you for your comment, we have adjusted some parts :

1. The objective has been repositioned in the introduction.
2. We have improved the methods section.
3. The term ‘recent’ has been changed to **“2017-2024.”**
4. Grammatical errors have been fixed in the method section

**Introduction**

Comment from reviewer :

 Authors could write introduction more crisply by avoiding repetition of enhancing patient comfort and improving outcomes; and reducing the content on adult population. It broadly could include what is available in literature about paediatric preoperative carbohydrate loading and end with what this review aims to achieve.

Respond:

1. We reduced content related to the adult population and add more pediatric content

**Methods**

Comment from reviewer;

1. The PRISMA-ScR reporting guidelines could be accessed at Open Science Framework platform. But the authors have not mentioned if the protocol for the review exists, registration of the protocol and the link to the same. 
2. A formal risk of bias assessment may not be necessary for scoping reviews. 
3. Future tense of methodology needs to be changed to past tense throughout the methods section. Ex. The selection process will involved, Full-text articles would be reviewed, etc.
4. Eligibility criteria – includes the word ‘recent’ the time duration of which has not been explained. 
5. The authors must provide truncations of the search results from various databases in a supplementary file. They could present the full electronic search strategy for at least 1 database, including any limits used, such that it could be repeated. Authors could include the date of database search.
6. Figure 1 PRISMA flow diagram mentions - Records excluded** Authors could mention  what two asterisks stand for in the footnote of the figure mentioning the reason for exclusion. Authors mention ‘Reports not retrieved’ but have not mentioned the reason for non-retrieval. Authors mention 240 reports excluded after assessing for eligibility from 365 reports. However only 10 articles are included in review. Authors need to mention the reasons for excluding 115 articles.
7. Clarity on how many reviewers contributed to data charting will be useful instead of mentioning ‘multiple’.

Respond:

 Thank you for your detailed feedback;

1. We have already included study protocol at OSF. Actually, we had added it prior submitting the manuscript but the editor asked us to remove the protocol file.
2. Thank you for your feedback. We agree that a formal risk of bias assessment may not be required for a scoping review. However, PRISMA-ScR guidelines recommend including a section for critical appraisal of the evidence. Although using RoB 2.0 is a formal approach, we believe it provides a thorough and reliable overview of study quality. Employing a trusted tool like RoB 2.0 allows us to gauge the clinical study quality in a structured way, enhancing the overall insight into the evidence included in our review.
3. Grammatical errors have been fixed.
4. The terms ‘recent’ and ‘multiple’ have been edited.
5. We have provided truncations of the search results from various databases in a supplementary file, including the full electronic search strategy for at least one database with specified limits and the date of the database search.
6. The PRISMA flowchart was revised to clarify the reasons for exclusion, noting that the asterisks in the footnote referred to a format picture that had not been deleted. Additionally, we included the reasons for excluding 385 articles from the initial assessment of 395 full-text articles.
7. The terms ‘recent’ and ‘multiple’ have been edited.

**Results**

Comment from reviewer;

1. Table 1 legends should include expansion of short forms used in the table.
2. Length of stay – though authors mention inconsistency in results, the results appear to be consistent in studies not demonstrating clinically relevant difference amongst the groups. This could be restructured.
3. Carvalho in their abstract mention - Four patients (21%) in the fasting group but none in the CHO group were hyperglycemic before surgery (p = 0.04). This has been missed while compiling this review.
4. We have found a few relevant studies which were not included in this review. Could authors provide an explanation for the same?

Gawecka A, Mierzewska-Schmidt M. Tolerance of, and metabolic effects of, preoperative oral carbohydrate administration in children - a preliminary report. Anaesthesiol Intensive Ther. 2014 Apr-Jun;46(2):61-4. 

 Bauer JM, Trask M, Coughlin G, Gopalan M, Gupta A, Yaszay B, Yang S, Grigg E. Pre-operative carbohydrate drink in pediatric spine fusion: randomized control trial. Spine Deform. 2024 Sep;12(5):1283-1287. 

Respond:

 Thank you for your thorough feedback;

1. We have added the expansions of all abbreviations and short forms used in Table 1 to the table legend, as suggested.
2. Regarding Length of Stay (LOS), we acknowledged the inconsistency in previous reports and agreed that the results should be restructured. The studies consistently showed no clinically meaningful difference in the length of hospital stay.
3. Preoperative hyperglycemia was added to the study characteristics table.
4. We excluded the study by Gawecka A et al. (2014) from our scoping review due to its publication year and added the study by Bauer JM et al. (2024).

**Discussion**

Comment from reviewer;

1. Authors mention that - Studies consistently demonstrated that PCL helps maintain higher yet stable blood glucose levels. However, Bharadwaj et al show lower glucose levels in children receiving PCL. The effect of PCL on glucose levels could be rewritten as inconsistent. Authors could highlight and explore the inconsistency in glucose levels after PCL and identify if any factors like duration of fasting affected it.
2. In Laird et al. difference of ketones by 0.1 and lactates by 0.3 mmol/L with overlapping ranges though may be statistically significant, the clinical relevance of such small differences may not be relevant. Authors could highlight such trivial ‘statistically significant’ results across all the articles included and explore the clinical relevance in the discussion of this review.
3. Authors could include a mention of recently published meta-analysis on the topic and compare results - Li Y, Chen L, Su Y, Zhang X. Preoperative Oral Carbohydrates for Children: A Meta-Analysis and Systematic Review. Biol Res Nurs. 2024 Oct;26(4):624-635. 
4. Scoping reviews can have the following biases: language, search, data extraction, reporting, reviewer, publication, and selection bias. Authors need to mention these in the limitations including steps taken to mitigate them.

Respond:

1. We revised our analysis to emphasize the inconsistencies in blood glucose levels following preoperative carbohydrate loading (PCL), highlighting factors such as fasting duration. Additionally, we addressed trivial statistically significant findings, such as the small differences in blood ketones and lactates reported by Laird et al., to explore their clinical relevance.
2. The current meta-analysis was added and discussed.
3. We already included Chen et al. study
4. We explained the limitations of the study
